# Mitogenome of a cryptic species within *Uropsilus* and divergence time estimation

**DOI:** 10.3109/19401736.2015.1060440

**Published:** 2017-10-19

**Authors:** Yu Xu, Yunting Hu, Feiyun Tu

**Affiliations:** a School of Life Sciences, Guizhou Normal University, Guiyang, China;; b Guizhou Normal University Library, Guizhou Normal University, Guiyang, China;; c Institute of Wildlife Conservation, Jiangxi Academy of Forestry, Nanchang, China

**Keywords:** Cryptic species, divergence time, mitogenome

## Abstract

*Uropsilus* sp. 4 is a new cryptic species, collected in Changyang county, Hubei province, China. In this study, the whole mitochondrial genome of *Uropsilus* sp. 4 was first determined and characterized. The genome is 16,542 bp in length, containing 13 protein coding genes, 22 transfer RNA genes, two ribosomal RNA genes, and a putative control region. Base on NJ, ML, and BI methods, we obtained the same topologies. *U.* sp. 4 clustered with *U. gracilis* and the divergence time was 1.78 Ma (95% CI 1.24–2.32 Ma), in concordance with the third period of last orogenic push of the Qinghai-Tibetan Plateau, might contribute to the speciation of *U*. sp. 4.

There are 10 recognized species within the genus *Uropsilus*: *U. aequodonenia*, *U. andersoni*, *U. atronates*, *U. gracilis*, *U. investigator*, *U. nivatus*, *U. sorcipes*, *U*. sp. 1, *U*. sp. 2, and *U*. sp. 3 (Liu et al. 2013; Wan et al. 2013). We collected a specimen, SAF_HB10N042, female, from Changyang county, Hubei province, China. By sequencing the cyt b gene and constructing a neighbor-joining (NJ) phylogenetic tree following the study of Wan et al. (2013), we identified this individual as a new cryptic species, *Uropsilus* sp. 4. To date, mitogenomes of six species of *Uropsilus* have been reported: *U. aequodonenia*, *U. andersoni*, *U. soricipes*, *U. nivatus*, *U*. sp.1, and *U. gracilis* (Tu et al. 2015; Hou et al. 2016). More mitogenomes are required to better know phylogenetic relationships within *Uropsilus*.

In this study, we thus determined the complete mitochondrial genome of *U.* sp. 4 and also built the phylogenetic trees with other eleven related species based on 12 protein-coding genes (PCGs) using the NJ, maximum likelihood (ML), and Bayesian inference (BI) methods. NJ analysis was performed by MEGA 6.06 (MEGA Inc., Englewood, NJ) (Tamura et al. 2013) based Kimura-2-parameter model. ML was carried out online software PhyML 3.0 (Bioo Scientific, Austin, TX) (Guindon et al. 2010) with default parameters. BI analysis was done by MrBayes 3.1.2 (MrBayes Inc., La Jolla, CA) (Ronquist and Huelsenbeck 2003) and the process parameters were based on the study of Tu et al. (2012). *Neotetracus sinensis* was set as outgroup. To estimate divergence time (with 95% confidential interval (CI)) between *U*. sp. 4 and its sister species, we performed the analysis using MEGA 6.06 (Tamura et al. 2013) based on the NJ tree of PCGs. Two calibration points were defined (Wan et al. 2013): (i) the oldest known Talpini was from the early Oligocene at approximately 33.9–32.6 million years ago (Ma); (ii) the oldest known *U. soricipes* was at 2.4–2.0 Ma.

The complete mitochondrial genome of *U.* sp. 4 (GenBank Accession Number KM503088, 16,542 bp), consisting of 13 protein-coding genes, 22 transfer RNA genes, two ribosomal RNA genes, and a displacement loop region (Table S1). Its genome structure is similar to other mammal mitogenomes (Chen et al. 2015; Tu et al. 2015; Wang et al. 2016). We generated the similar topologies (Figure 1). Within the genus *Uropsilus*, *U*. sp. 4 clustered with *U. graclis*. Divergence time between *U.* sp. 4 and *U. gracilis* was 1.78 Ma (95% CI 1.24–2.32 Ma). *U. gracilis*, collected in the Jinfo Mountains, Chongqing (Hou et al. 2016), which is located in the eastern Qinghai-Tibetan Plateau (QTP). The divergence time between *U.* sp. 4 and *U. gracilis* is in concordance with the third phase of last orogenic push of the QTP occurring about 1.7 Ma (Li et al. 2001). Therefore, the uplifted event might contribute to the speciation of *U*. sp.4. However, further study needs to confirm this.

**Figure 1.**
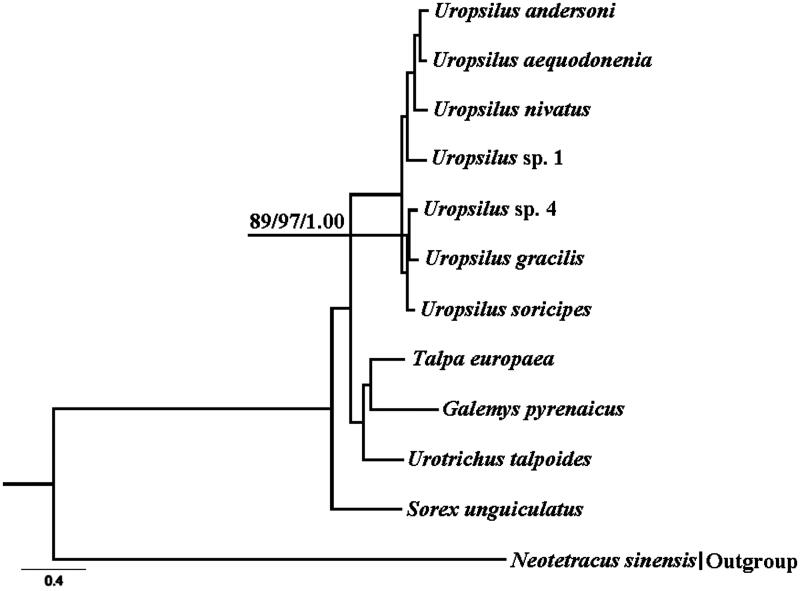
Bayesian 50% majority-rule consensus phylogenetic tree based on Bayesian analysis of 12 protein-coding genes. *Neotetracus sinensis* was used as an outgroup. The numbers on the internode branches from left to right were the NJ, ML and BI support values, respectively. Sequence data used in the study are the following: *Uropsilus* sp. 4 (KM503088), *Uropsilus andersoni* (JX945573), *Uropsilus aequodonenia* (KC516778), *Uropsilus nivatus* (JX945574), *Uropsilus* sp. 1 (JX034737), *Uropsilus gracilis* (KM379136), *Uropsilus soricipes* (JQ658979), *Talpa europaea* (NC_002391), *Galemys pyrenaicus* (NC_008156), *Urotrichus talpoides* (NC_005034), *Sorex unguiculatus* (NC_005435), and *Neotetracus sinensis* (JX519466).

## Supplementary Material

TMDN_A_1060440_Supplementary_Information.zip
