# Supplementary material for: Mitogenome of a cryptic species within Uropsilus and divergence time estimation
Source: Mitochondrial DNA B Resour. 2017 Oct 19;2(2):685–6. doi: 10.3109/19401736.2015.1060440 (PMC7800900; doi:10.3109/19401736.2015.1060440)
Supplement: TMDN_A_1060440_Supplementary_Information.zip [file TMDN_A_1060440_SM2083.zip › Table S1.doc]

|  | Position | |  | Codon | |  |  |  |
| --- | --- | --- | --- | --- | --- | --- | --- | --- |
| Gene | From | To | Size (bp) | Start | Stop | Strand | Anticodon | Intergenic nucleotide |
| tRNA-Phe | 1 | 68 | 68 |  |  | H | GAA | 0 |
| 12S rRNA | 69 | 1033 | 965 |  |  | H |  | -2 |
| tRNA-Val | 1032 | 1099 | 68 |  |  | H | TAC | 0 |
| 16S rRNA | 1100 | 2683 | 1584 |  |  | H |  | 0 |
| tRNA-Leu(UUR) | 2684 | 2759 | 76 |  |  | H | TAA | 2 |
| Nd1 | 2762 | 3718 | 957 | ATG | TAG | H |  | -2 |
| tRNA-Ile | 3717 | 3785 | 69 |  |  | H | GAT | -3 |
| tRNA-Gln | 3783 | 3855 | 73 |  |  | L | TTG | 1 |
| tRNA-Met | 3857 | 3925 | 69 |  |  | H | CAT | 0 |
| Nd2 | 3926 | 4972 | 1047 | ATA | TAG | H |  | -2 |
| tRNA-Trp | 4971 | 5038 | 68 |  |  | H | TCA | 4 |
| tRNA-Ala | 5043 | 5111 | 69 |  |  | L | TGC | 1 |
| tRNA-Asn | 5113 | 5185 | 73 |  |  | L | GTT | -10 |
| OL | 5176 | 5232 | 57 |  |  | L |  | -8 |
| tRNA-Cys | 5225 | 5288 | 64 |  |  | L | GCA | 0 |
| tRNA-Tyr | 5289 | 5357 | 69 |  |  | L | GTA | 1 |
| Cox1 | 5359 | 6903 | 1545 | ATG | TAG | H |  | 1 |
| tRNA-Ser(UCN) | 6905 | 6973 | 69 |  |  | L | TGA | 7 |
| tRNA-Asp | 6981 | 7049 | 69 |  |  | H | GTC | 1 |
| Cox2 | 7051 | 7734 | 684 | ATG | TAA | H |  | 3 |
| tRNA-Lys | 7738 | 7804 | 67 |  |  | H | TTT | 1 |
| Atp8 | 7806 | 8009 | 204 | ATG | TAA | H |  | -43 |
| Atp6 | 7967 | 8647 | 681 | ATG | TAA | H |  | -1 |
| Cox3 | 8647 | 9430 | 784 | ATG | T-- | H |  | 1 |
| tRNA-Gly | 9432 | 9500 | 69 |  |  | H | TTC | 0 |
| Nd3 | 9501 | 9846 | 346 | ATA | T-- | H |  | 0 |
| tRNA-Arg | 9847 | 9915 | 69 |  |  | H | TCG | 0 |
| Nd4L | 9916 | 10212 | 297 | ATG | TAA | H |  | -7 |
| Nd4 | 10206 | 11576 | 1371 | ATG | TAA | H |  | 1 |
| tRNA-His | 11578 | 11646 | 69 |  |  | H | GTG | 0 |
| tRNA-Ser(AGY) | 11647 | 11706 | 60 |  |  | H | TGA | 3 |
| tRNA-Leu(CUN) | 11710 | 11779 | 70 |  |  | H | TGA | 0 |
| Nd5 | 11780 | 13600 | 1821 | ATT | TAA | H |  | 3 |
| Nd6 | 13584 | 14111 | 528 | ATG | TAA | L |  | 0 |
| tRNA-Glu | 14112 | 14180 | 69 |  |  | L | TTC | 4 |
| Cytb | 14185 | 15324 | 1140 | ATG | AGA | H |  | 0 |
| tRNA-Thr | 15325 | 15392 | 68 |  |  | H | TGT | 2 |
| tRNA-Pro | 15395 | 15461 | 67 |  |  | L | TGG | 0 |
| D-loop | 15462 | 16542 | 1081 |  |  | H |  |  |

Table S1 Mitogenome stucture of *Uropsilus* sp. 4
